# Comprehensive Enhancement of Prepolymer-Based Flexible Polyurethane Foams’ Performance by Introduction of Cost-Effective Waste-Based Ground Tire Rubber Particles

**DOI:** 10.3390/ma15165728

**Published:** 2022-08-19

**Authors:** Wiktoria Żukowska, Paulina Kosmela, Paweł Wojtasz, Mariusz Szczepański, Adam Piasecki, Roman Barczewski, Mateusz Barczewski, Aleksander Hejna

**Affiliations:** 1 Department of Polymer Technology, Gdańsk University of Technology, Narutowicza 11/12, 80-233 Gdańsk, Poland; 2 Institute of Materials Engineering, Poznan University of Technology, Jana Pawła II 24, 60-965 Poznan, Poland; 3 Institute of Applied Mechanics, Poznan University of Technology, Jana Pawła II 24, 60-965 Poznan, Poland; 4 Institute of Materials Technology, Poznan University of Technology, Piotrowo 3, 61-138 Poznan, Poland

**Keywords:** polyurethane foam, ground tire rubber, composites, prepolymer, recycling

## Abstract

Material innovations in polyurethane (PU) foams should ideally combine performance enhancement, environmental impact limitation, and cost reduction. These goals can be achieved by applying recycled or waste-based materials without broader industrial applications, implicating their low price. Herein, from 5 to 20 parts by weight of ground tire rubber (GTR) particles originated from the recycling of postconsumer car tires were incorporated into a flexible foamed PU matrix as a cost-effective waste-based filler. A two-step prepolymer method of foams manufacturing was applied to maximize the potential of applied formulation changes. The impact of the GTR content on the foams’ processing, chemical, and cellular structure, as well as static and dynamic mechanical properties, thermal stability, sound suppression ability, and thermal insulation performance, was investigated. The introduction of GTR caused a beneficial reduction in the average cell diameter, from 263.1 µm to 144.8–188.5 µm, implicating a 1.0–4.3% decrease in the thermal conductivity coefficient. Moreover, due to the excellent mechanical performance of the car tires—the primary application of GTR—the tensile performance of the foams was enhanced despite the disruption of the cellular structure resulting from the competitiveness between the hydroxyl groups of the applied polyols and on the surface of the GTR particles. The tensile strength and elongation at break were increased by 10 and 8% for 20 parts by weight GTR addition. Generally, the presented work indicates that GTR can be efficiently applied as a filler for flexible PU foams, which could simultaneously enhance their performance, reduce costs, and limit environmental impacts due to the application of waste-based material.

## 1. Introduction

Polyurethane (PU) foams are widely applied in multiple branches of industry, including bedding, furniture, building, construction, and automotive sectors [1]. They account for over half of the total PU market, so their development aimed at performance enhancement, price reduction, and limiting the environmental impacts is essential in a broader perspective [2,3]. The perfect innovations should combine these aspects to maximize the application potential and benefits for future users and the environment. In the case of PU foams, the novel solutions could include modifications of currently applied production and processing techniques or substituting conventionally applied raw materials with new alternatives, either from renewable or waste-based resources [4]. Significantly more affordable and innovation absorbing is the second direction related to the substation of conventional petroleum-based raw materials for PUs.

Over the years, the main direction has been the application of plant oils or their derivatives in biopolyols manufacturing [5]. Biopolyols are usually obtained from plant oils, which are available in a particular region: for Europe, rapeseed and sunflower oils [6,7]; for Asia, palm and coconut oil [8]; for North America, soybean oil [9]. Multiple processes have been developed to convert plant oils into biopolyols, but they all have one disadvantage. They are mainly using edible vegetable oils, whose broader application in PU manufacturing may adversely affect their price and cause an increase in the prices of various food products [10,11]. Moreover, the application of virgin vegetable oils in biopolyols manufacturing, despite the benefits related to the reduced global warming potential compared to their petroleum analogs, implicates relatively high environmental impacts related to the inputs of crop production, including seeds, fertilizers, plant protection products, and land and water use [12,13].

Therefore, lately, more emphasis has been placed on using waste-based materials as PU components, either as raw materials for biopolyols’ production or as fillers, which could be introduced into the PU matrix and reduce the use of virgin material [14,15,16,17,18]. Ideally, the application of waste-based material should not only provide an effective method for its utilization but also enhance the performance of the unfilled matrix. Superior selected properties should characterize such material compared to the polymer matrix, e.g., higher strength or thermal stability, reduced flammability or smoke generation, and higher conductivity. In the case of solid materials, mechanical enhancement may be achieved by incorporating various types of waste fibers, including natural ones [19,20,21]. Thermal stability enhancement or reduced flammability may be induced by the application of various waste-based inorganic fillers, e.g., fly ash [22], red mud [23], copper slag [24] or steel slag [25], as well as organic ones like chicken feather [26], nut shells [27,28], or leather [29]. Conductivity can be boosted by the incorporation of waste-based metal particles [30,31] or char obtained from plant-based wastes [32,33].

Besides the above-mentioned conventional waste fillers based on lignocellulose or inorganic mineral materials, plastic wastes can be recycled and introduced into virgin polymer matrices. Such an approach creates the utilization methods and provides new features to the prepared composites resulting from the properties of primary materials, such as reduced specific gravity due to the incorporation of foamed plastics [34], reduced flammability related to the use of phenolic foam or poly(vinyl chloride) residues [35,36], or enhanced toughness attributed to the incorporation of rubber particles [37,38]. Considering waste rubber, the market offers an excellent opportunity for using postconsumer car tires. They are generated in massive amounts irrespectively of the location and are considered problematic waste. Due to their composition and related toxic effects, they should not be combusted without proper complex and expensive apparatus. Moreover, such an approach enables only 40% recovery of the energy used for their production [39]. Material recycling yielding ground tire rubber (GTR) poses a significantly more beneficial alternative. To improve its profitability and popularize it, there is a need for the potential industrial applications of GTR, so it is justified to investigate its incorporation into polymer composites. Until now, GTR has been investigated as filler in the construction of tunnels, base layers of road surfaces, and sports surfaces, as well as for manufacturing car mats, windshield wipers, cattle mats, or asphalt modifiers [40]. However, these applications still do not guarantee sufficient demand for processed rubber waste. Therefore, a great deal of research is currently underway to find potential applications for GTR in the plastics industry. Incorporating GTR into PU matrices has an undeniable economic advantage because its price is at least ten times lower than PU systems [41].

In our previous works, we applied GTR as a filler for foamed PU composites [42,43,44,45,46,47]. Foams contained unmodified GTR, thermomechanically reclaimed with or without the addition of the oils and chemically oxidized. Nevertheless, in most cases, despite the selected beneficial effects, the disruption of the cellular structure was noted irrespectively in the applied GTR treatments, which limited the overall composite performance. Similar effects were noted by other researchers [48,49,50]. However, in all of the works mentioned above, the foams were obtained using the single-step method of PU preparation. None of the research works reported the preparation of foamed PU/GTR composites using the second method of PU preparation—the prepolymer method, which is applied and beneficial in the case of the increased viscosity of polyol mixture [51], which was observed in previous works after GTR incorporation [44,46].

Therefore, the presented work was aimed at a comprehensive investigation of the performance of foamed flexible PU/GTR composites obtained by the two-step prepolymer method. The impact of the introduced rubber particles on foams’ processing, chemical, and cellular structure, as well as static and dynamic mechanical properties, thermal stability, sound suppression ability, and thermal insulation performance, was analyzed.

## 2. Materials and Methods

### 2.1. Materials

**Figure 1:**
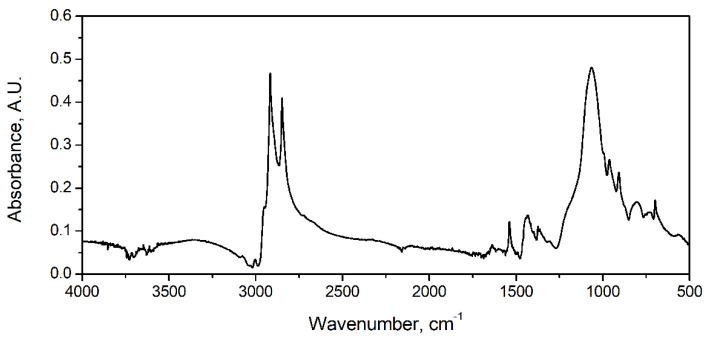
FTIR spectra of GTR applied as filler for investigated PU-based composites.

Table 1 provides the details on the materials applied in the presented study. Moreover, Figure 1 presents the FTIR spectra of the applied GTR. Most of the observed signals are associated with the different vibrations of the C-H bonds included in various chemical groups. Peaks at 2848 and 2916 cm^−1^ are characteristic for the symmetric and asymmetric stretching vibrations of the C-H bonds in the methylene groups present in the rubber macromolecules. The band at 1542 cm^−1^ points to the stretching of C=C unsaturated bonds present in the benzene rings included in the structure of styrene–butadiene rubber [52]. Signals at 1432 and 1373 cm^−1^ can be attributed to the deformation vibrations of the C-H bonds in the C=CH_2_ and CH_3_ groups, respectively [53]. The noticeable peak at 1063 cm^−1^ points to the vibrations of the C-O-C bonds. Smaller shoulder bands at 995 and 901 cm^−1^, as well as minor peaks at 801, 733, and 701 cm^−1^, are characteristic for C-O, C-C, C-H, S=O, and C-S bonds [52].

### 2.2. Preparation of Flexible Polyurethane Foams

PU foams were prepared on a laboratory scale by a two-step method. The first step was the preparation of the prepolymer. Poly(tetramethylene ether)glycol (PTMG) was dehydrated at 90 °C under vacuum for 90 min. Subsequently, the calculated amount of toluene diisocyanate (TDI) was introduced, and synthesis was carried out at 60 °C under a vacuum for 120 min. The reaction was carried out in a 1 L reactor with a three-necked lid and a connected Rocker Tanker 230 vacuum pump. The content of the unreacted isocyanate groups in the prepolymer was 18.20%, measured using method for the determination of free isocyanate group content by titration with dibutylamine, according to ASTM D-2572. Next, the blend, including PTMG, glycerol, catalysts, foam stabilizer, blowing agent, and, in the case of composite foams, GTR, was prepared. In the case of composite foams, GTR in the amount of 5–20 parts by weight (pbw) of the PU system was homogenized with PTMG and glycerol for 30 s at 500 rpm prior to the incorporation of other components. When the complete blend was prepared, it was mechanically mixed with a previously prepared prepolymer for 10 s at 1000 rpm and poured into a closed aluminum mold heated up to 60 °C. After demolding, samples were held at 60 °C for 24 h and conditioned at room temperature (21–23 °C) for another 24 h. Moreover, for the analysis of foaming kinetics, additional samples were prepared without molding and left for the free rise. Table 2 contains the details of foam formulations.

### 2.3. Measurements

The following processing times of the prepared PU-based composites were measured: rise time (time of the end of volumetric expansion) and the tack-free time (time until the surface stopped being tacky to the touch). Moreover, during polymerization, the temperature of the foam core was measured using a thermocouple. The temperature was checked every 2 s.

After conditioning, foamed PU composites were analyzed following the standard procedures.

The color of the unfilled PU foam and foamed PU/GTR composites was evaluated following the recommendations of International Commission on Illumination (CIE) [54]. Applied system consists of three color components: L*—lightness (L* = 0 for black and L* = 100 for white), a*—the green(−)/red(+) component, b*—the blue(−)/yellow(+) component. For each sample, 15 specimens were analyzed. The color was determined by optical spectroscopy using Hunter Associates Laboratory, Inc. (Reston, VA, USA) Miniscan MS/S-4000S spectrophotometer, placed in a specially designed light trap chamber to eliminate the impact of the external light sources. The following color parameters were determined:

- total color difference parameter (ΔE*), calculated according to the Equation (1) [55]:

ΔE* = [(ΔL*)^2^ + (Δa*)^2^ + (ΔL*)^2^]^0.5^ (1)

- chroma (C*_ab_), calculated according to the Equation (2):

C*_ab_ = [(a*)^2^ + (b*)^2^]^0.5^ (2)

- hue (h_ab_), calculated according to the Equation (3):

h_ab_ = tan^−1^ (b*/a*) (3)

Determined color parameters were also converted to the commonly used Adobe RGB color space [56].

The apparent density of the prepared composites was determined according to PN-EN ISO 845 standard. The cube-shaped samples were measured with a slide caliper with an accuracy of 0.1 mm and weighed using an analytical balance with an accuracy of 0.0001 g.

The chemical structures of the prepared samples were determined using Fourier transform infrared (FTIR) Nicolet Spectrometer IR200 from Thermo Scientific (Waltham, MA, USA). The device was equipped with an ATR attachment with a diamond crystal. Measurements were performed with 1 cm^−1^ resolution in the range from 4000 to 400 cm^−1^ and 64 scans.

The cellular structure of the prepared PU/GTR composites was investigated with a scanning electron microscope (SEM) MIRA3 from Tescan (Brno, Czech Republic). Analyzed samples were carbon-coated with a Jeol JEE 4B vacuum evaporator from Jeol USA (Peabody, MA, USA). The thickness of the coating was approximately 20 nm. The accelerating voltage of 5 kV and secondary electron detector were used.

The images obtained with SEM microscopy were analyzed with ImageJ software. The following shape descriptors of cells were determined:

Circularity (C), calculated according to the Equation (4):

R = (4 ∙ π ∙ A)/P^2^ (4)

Aspect ratio (AR), calculated according to the Equation (5):

AR = L_L_/L_S_ (5)

Roundness (R), calculated according to the Equation (6):

R = (4 ∙ A)/(π ∙ L_L_^2^) (6)

where P is the perimeter, L_L_ and L_S_ are the lengths of the longer and shorter axis of the fitted ellipse, and A is the area of the fitted ellipse.

The content of open cells in manufactured materials was determined with Ultrapyc 5000 Foam gas pycnometer from Anton Paar (Graz, Austria). Following measurement settings were applied: gas—nitrogen; gas pressure—3.0 psi; measurement type—corrected; flow direction—sample first; target temperature—20.0 °C; flow mode—monolith; cell size—medium, 45 cm^3^; preparation mode—flow, 0.5 min.

The thermal conductivity coefficient (λ) of the obtained materials was determined using the heat flow meter HFM 446 from Netzsch (Selb, Germany). Samples with a thickness of 4 cm were tested in the temperature range from 1 to 19 °C using the average temperature of 10 °C.

The sound absorption coefficients of the material samples were determined following the ISO 10534-2 [57] and ASTM E1050-8 [58] standards. The following equipment was used to carry out the tests: two BSWA impedance tubes (SW422 and SW477), MC 3242 data acquisition hardware, PA50 power amplifier, BSWA VA LAB4 software (produced by BSWA-Technology Co., Ltd., Beijing, China), and two MI 19 microphones—1/4 inch IEPE standard (produced by Roga Instruments, Nentershausen, Germany). The measuring system was calibrated with a CA114 acoustic calibrator (BSWA Technologies Co., Ltd., Beijing, China). The LB−575 climate meter (produced by LAB-EL, Reguły, Poland) monitored atmospheric pressure, temperature, and air humidity. The preparation of the samples for testing included cutting out from the base material (approximately 22 mm thick) and discs with a diameter of 30 and 100 mm. The samples were tested, removing about 6 mm of the uneven upper layer. This treatment was also aimed at exposing the internal cellular structure of the material (foam). After cutting off the top layer, the samples were 16 mm thick.

The composites’ tensile strength was evaluated following ISO 1798 standard. The beam-shaped samples with 10 × 10 × 100 mm^3^ dimensions subjected to static tensile tests, which were conducted using Zwick/Roell tensile tester (Ulm, Germany) at a constant speed of 500 mm/min.

Dynamical mechanical analysis (DMA) was performed using a Q800 DMA instrument from TA Instruments (New Castle, DE, USA) at a heating rate of 4 °C/min and the temperature range from −100 to 150 °C. Samples were cylindrical-shaped, with dimensions of 10 × 12 mm.

The thermogravimetric (TGA) analysis was performed using the TG 209 F3 apparatus from Netzsch (Selb, Germany). Samples weighing approx. 10 mg were placed in a ceramic dish. The study was conducted in a nitrogen atmosphere from 30 to 800 °C with a temperature increase rate of 10 °C/min.

## 3. Results and Discussion

**Figure 2:**
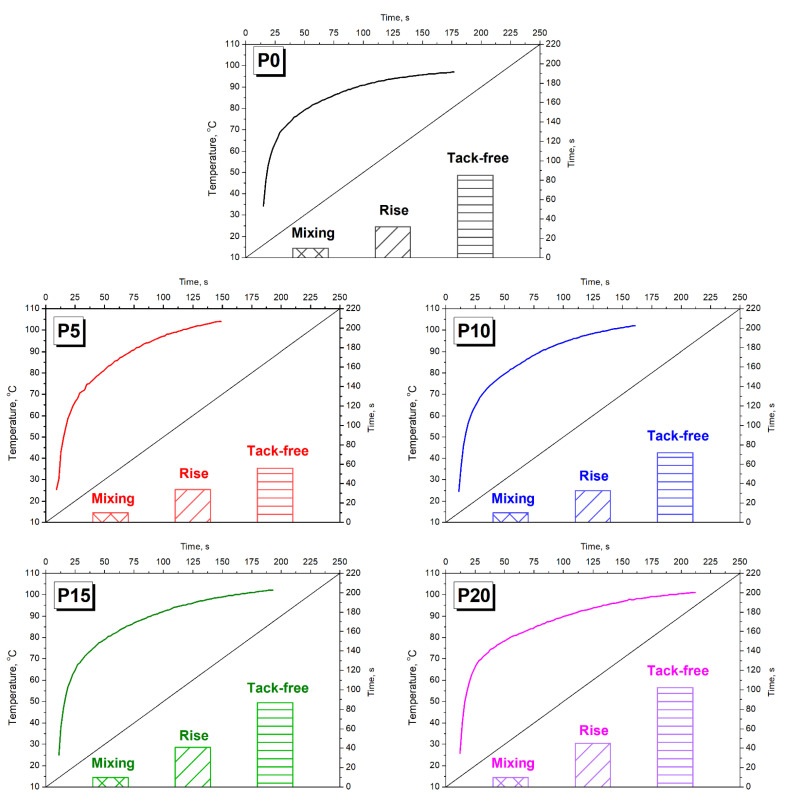
The impact of GTR addition on the processing times (shaded areas) and temperature (curves) reached by the core of foam during volumetric expansion.

Figure 2 presents the influence of the GTR content on the foaming kinetics of the prepared composite foams. Significant differences were noted in processing times, which can be attributed to the introduction of solid particles into the polyol mixture. For the unfilled foam, the rise time was 32 s, while the addition of 20 pbw of GTR resulted in its elongation to 45 s. Such a phenomenon was attributed to the increase in the polyol mixture’s viscosity induced by solid particles. This effect is commonly observed in the case of composite PU foams [1]. Moreover, the PU structure has been weakened because of the disrupted balance between hydroxyl and isocyanate groups present in the PU system. Applied GTR particles, particularly hydroxyl groups present on their surfaces, as proven in our previous works [47,59], partially attracted isocyanate groups and reduced their amount taking part in PU polymerization. A similar effect was noted for the tack-free time, which was noticeably elongated for the highest GTR contents due to the competitivity between hydroxyl groups present in the reacting mixture [46].

Surprisingly, for the lowest contents of GTR, foaming was accelerated compared to neat PU. Such an effect could be associated with the nucleating activity of solid rubber particles. During PU foaming, before beginning the volumetric expansion, the reacting mixture of the polyols and isocyanates must reach supersaturation with blowing gas. Afterward, the nucleation begins and foam rises [60], so the GTR nucleating activity leads to more rapid foaming. Apparently, for the lower contents of GTR, the additional nucleating activity effect overpowered the viscosity increase, which contradicts and limits the foaming rate.

Considering the maximum temperature reached by the foams’ core during polymerization, it was slightly higher for composite foams (101–104 °C) than for the reference sample (97 °C). The highest value was noted for the P5 foam, related to the fastest polymerization and foaming. Therefore, the heat dissipation by convection to the surrounding environment was hindered due to more rapid heat build-up caused by exothermic chemical reactions.

**Figure 3:**
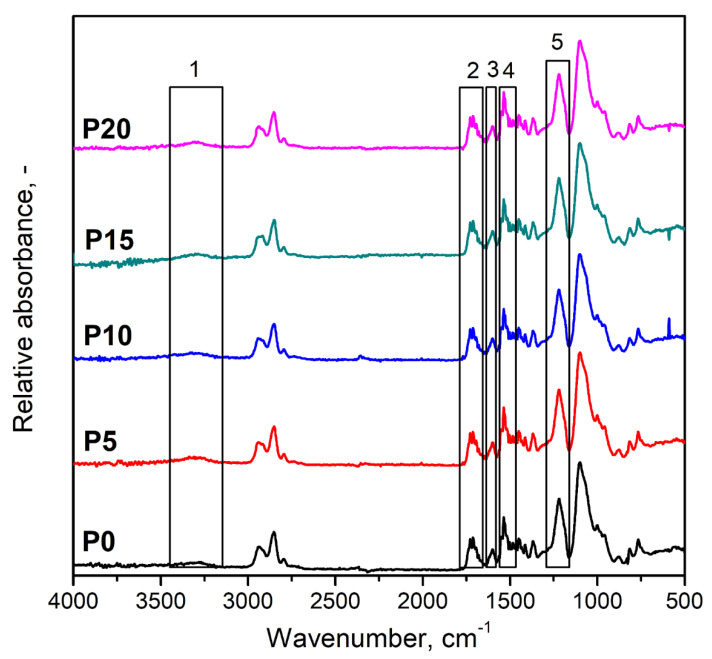
FTIR spectra of prepared PU/GTR composite foams and the unmodified PU foam.

Figure 3 presents the FTIR spectra of unfilled foamed PU matrix and prepared PU/GTR composites. Qualitatively, all spectra show a very similar appearance, indicating a lack of significant changes in the chemical structure induced by GTR incorporation. Signals marked with the numbers 1–5 indicate the presence of bonds characteristic of PU materials. Signals (1) at 3230–3370 cm^−1^ were attributed to stretching vibrations of N-H bonds, while peaks (3) around 1598 cm^−1^ and (4) at 1515–1530 cm^−1^ were related to the bending N-H vibrations [61,62]. Carbonyl bonds C=O can be recognized by the presence of peaks characteristic of their stretching vibrations at 1700–1735 cm^−1^ [63]. Signals (5) at 1220–1225 cm^−1^ are typical for stretching vibrations of C-N bonds [64]. The signals mentioned above confirm the efficient generation of urethane bonds during PU foams’ preparation. Other notable absorption bands were noted at 2860–2950 cm^−1^ and 1010–1120 cm^−1^ and were attributed to the presence of C-H and C-O bonds, respectively. Their presence was associated with the chemical structure of components used for manufacturing PU matrix and introduced GTR particles.

Table 3 presents the impact of the introduced filler on the appearance of the foams in quantitative terms. Due to the introduction of dark, black rubber particles, the parameter affected most significantly was foams’ lightness. It decreased proportionally to the content of the GTR in the composite foams. A significant decrease, even for 5 wt% content of GTR, was noted. Other color parameters were noticeably less influenced by the filler incorporation, which is attributed to the fact that, irrespectively of composition, the color of all samples could be described as gray. Gray colors are characterized by the low values of a*, b*, and chroma [65,66]. For such low chroma values, the influence of hue angle on the actual color of the material is negligible [67]. Moreover, the numerical data presented in Table 3 are related to the average color of the whole material. Actual foam images presented in Table 3 show that the appearance of the material is not homogenous, which is associated with the micrometric size of applied GTR particles. Nevertheless, the appearance of composite foams is in line with the appearance of PU/GTR foams presented in previous work [45].

**Figure 4:**
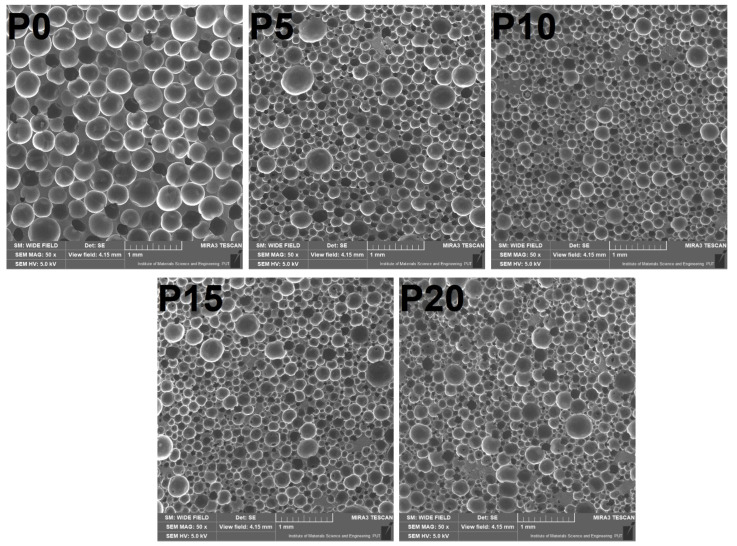
Images showing the cellular structure of prepared samples obtained with scanning electron microscopy.

Table 4 shows the parameters describing the cellular structure of prepared foams, whose images made with scanning electron microscopy are presented in Figure 4. It can be seen that the introduction of GTR particles into foamed PU matrix caused noticeable changes in cellular structure. The most crucial change was associated with the reduction in foams’ average cell diameter, one of the main parameters describing the foams’ structure, critical for their performance, and essential applications like thermal and acoustic insulation [68,69]. It can be observed in composite PU foams when filler particles act as nucleating agents [70,71]. Such an effect was noted in our previous work of flexible PU foamed composites filled with GTR [47]. Nevertheless, GTR particles may disrupt the cellular structure for higher loadings, which can be associated with the changes in the viscosity of the reacting mixture during polymerization and the insufficient surface area of the rubber particles [46]. Disruption of cellular structure can also be associated with changes in cells’ shape. The increasing filler content caused an increase in cells’ aspect ratio, indicating they were more ellipsoidal than unfilled foam. It can be attributed to the presence of filler particles, which increase the viscosity of the reaction mixture and increases the structure heterogeneity [72]. Similar effects have been noted by other researchers [73]. Roundness, as the antagonist of aspect ratio according to Equations (2) and (3), decreased with the GTR content, meaning the cells in the composite foams were less similar to a perfect circle compared to the unfilled matrix. Another parameter describing cells’ shape is circularity, which, compared to roundness, includes the aspect ratio between perpendicular diameters and the perfection of cells’ perimeter [74]. Therefore, its values are lower than roundness, decreasing significantly with the loading of GTR particles. Such an effect is related to the imperfections of cells’ perimeters at the interface with filler particles.

Besides the parameters describing the shape of cells, another important parameter describing the cellular structure of PU foams is the open cell content, which is critical for the many applications of these materials [75]. The opening of the cells due to the GTR incorporation could be attributed to the reduced strength of cells, which were not strong enough to keep foaming gas inside closed cells [76]. Again, such an effect can be attributed to the partial attraction of isocyanate groups by hydroxyl functionalities present on the surface of the GTR particles, which reduced the strength of the PU phase.

Structural changes in the PU foams induced by the GTR incorporation also affected the apparent density of the composites. For lower contents, a slight drop in this parameter was noted despite the presence of solid particles due to their nucleating activity [77]. Similar to changes in foaming kinetics, the additional nucleating activity of the GTR overpowered the increase of polyols’ mixture viscosity, so the enhanced nucleation reduced composites’ apparent density. Despite the nucleating activity for higher contents of GTR, the viscosity of the reacting mixture was apparently too high, which, combined with the above-mentioned reduced strength of the PU phase, limited the volumetric expansion and increased the apparent density.

Table 4 presents the sol fraction content values determined during swelling of the foams in toluene. It can be seen that they are increasing with the GTR content in the composites. For the unfilled PU foam sol fraction content, related to the presence of a noncrosslinked portion of the material, which can be extracted during swelling, it equaled 1.35 wt%, indicating slight imperfections in the balance between the isocyanate and hydroxyl groups present in the system. For the PU/GTR composites, the value of this parameter increased to 1.91–2.74 wt%, which can be attributed to the partial decomposition of the rubber network during the shredding of the postconsumer car tires aimed at the production of GTR. Similar effects were noted in our previous works on PU composites filled with GTR particles [46,47].

The above-mentioned parameters of the cellular structure have a significant influence on the thermal conductivity of the cellular materials, as thermal insulation is one of their primary applications. The precise, quantitative impact of the structural parameters on the thermal conductivity coefficient (λ) is very complex due to the dual nature of foamed materials and different heat transfer mechanisms. The most critical parameter is the apparent density, which quantifies the share of solid and gas phases in foamed materials due to the significant differences in λ values between solid PU, 200–250 mW/(m·K) reported by most studies [78,79], and gases present inside closed or open cells, from ~10 mW/(m·K) for hydrofluorocarbons to 12–15 mW/(m·K) for C_5_ hydrocarbons and carbon dioxide, to 25 mW/(m·K) for air [80,81].

Other structural parameters, cell size, and open cell content are more critical for heat exchange via radiation and convection. Increasing the cell size significantly enhances the radiative heat transfer and area of heat convection, so thermal insulation materials should be characterized by possibly small cells [82,83]. The high content of open cells facilitates temperature gradient-induced gas flow, simultaneously enhancing the convective heat transfer [84].

Considering the incorporation of solid GTR particles into prepared foams, they should replace the solid PU part in the material. Therefore, assuming a lower thermal conductivity coefficient of GTR compared to 200–250 mW/(m·K) reported by most studies for solid PU [78,79], the addition of GTR seems beneficial for insulation performance [85].

The above-mentioned theoretical considerations have been somehow confirmed for prepared PU/GTR composite materials. It can be seen that the incorporation of GTR resulted in a slight decrease in thermal conductivity coefficient compared to the unfilled foam. Such an effect should be considered beneficial because multiple works pointed to the disruption of the cellular structure due to GTR incorporation, which unfavorably affects the insulation performance [43,44,45,46,86]. The slight reduction of λ values could be attributed to the reduction of average cell diameters resulting from the nucleating activity of solid GTR particles and the increased polyol mixture viscosity. Nevertheless, increasing the content of open cells and the higher apparent density provide contradictory effects. Therefore, despite the significantly finer cellular structure, only minor changes in the thermal conductivity coefficients were noted.

**Figure 5:**
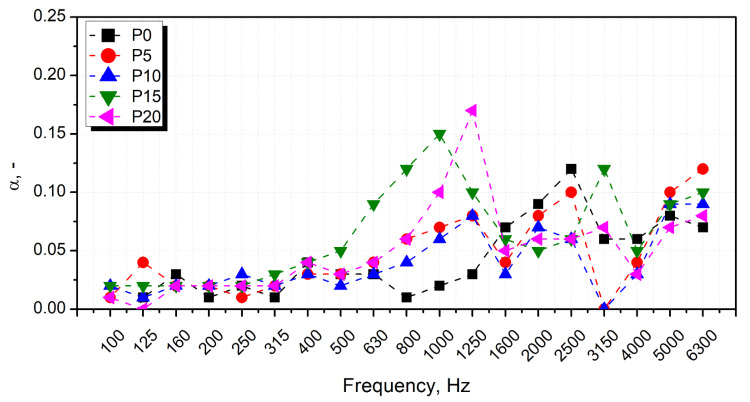
Sound absorption coefficient characteristics prepared samples in 1/3 octave bands (100–6300 Hz).

**Figure 6:**
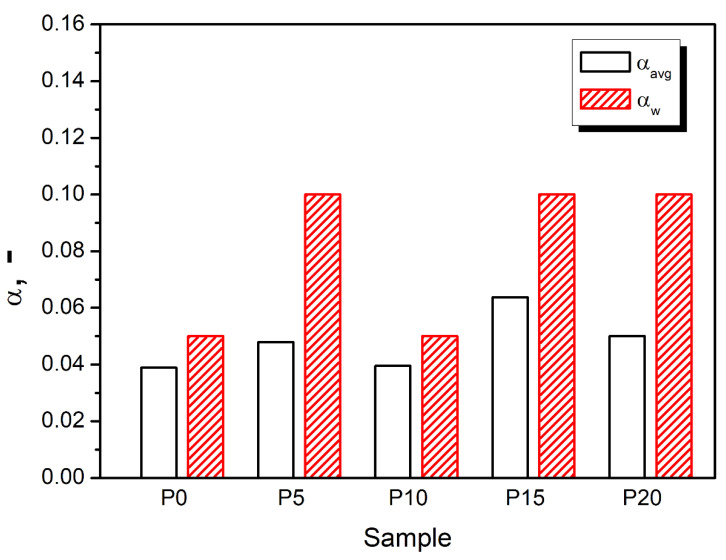
The average value of the sound absorption coefficient α_avg_ and the weighted sound absorption coefficients α_w_ of PU foams depending on GTR content.

The results of the sound absorption test are presented in Figure 5 in the form of characteristics containing the values of sound absorption coefficients in 1/3 octave bands (100–6300 Hz). The characteristics were created based on partial results obtained in the bands 63–500 Hz and 250–1600 Hz (using an impedance tube SW422 with a diameter of 100 mm and different spacing of microphones) and in the band 1000–6300 Hz (using a tube SW 477 with a diameter of 30 mm). The final result for each sample is the result of averaging three measurements. In addition, for each type of material (samples), the average value of the sound absorption coefficient α_avg_ and the weighted sound absorption coefficient α_w_ were determined (Figure 6). The value of α_avg_ was calculated according to the Equation (7):

α_avg_ = 1/n · ∑_(i = 1)_^n^ · α_f(i)_ (7)

where α_f(i)_ are the sound absorption coefficients (at the center frequencies f(i) from 100 Hz to 6.3 kHz) and n is the number of 1/3 octave bands.

Weighted sound absorption coefficients α_w_ were determined following the ISO 11654 standard. The basis for determining α_w_ are the practical sound absorption coefficients α_p(i)_ and the reference curve (specified in the standard), which are placed on one diagram. Values of α_p(i)_ are calculated in octave bands from 125 Hz to 4 kHz by averaging sound absorption coefficients in the 1/3 octave bands (arithmetic mean value of the three 1/3 octave sound absorption coefficients within the octave). Next, the reference curve is shifted in steps of 0.05 towards the measured values α_p(i)_ until the sum of the unfavorable deviations is less than or equal to 0.10. An unfavorable deviation occurs when the measured value is less than the value of the reference curve. Only these deviations are taken into account in the calculations. The weighted sound absorption α_w_ is defined as the value of the shifted reference curve at 500 Hz.

Considering sound absorption performance of cellular materials, the most important are the parameters quantitatively describing the structure, such as the average cell diameter and open cell content. The prepared foams presented behavior typical for such materials, with the sound absorption coefficient increasing with the sound frequency [87]. Such an effect is associated with the decreasing wavelength and facilitates the penetration of the PU cellular structure by the sound waves. Compared to the unfilled PU matrix, introducing the GTR particles slightly enhanced the α_avg_ values, indicating the increased damping ability. Other researchers noted similar effects due to the excellent damping performance of rubber [48,49]. Among the prepared composites, sample P10 is characterized by the lowest value of sound absorption coefficient, which can be attributed to the smallest cell size and open cell content limiting the sound attenuation effect [88]. The closed-cell structure has a limited ability to absorb sound waves, which has been reported in the literature [89,90]. The sound absorption performance of foams is also affected by their mechanical properties. The results of the dynamic mechanical analysis presented in the following sections point to the reduced damping ability of the cellular structure, limiting the conversion of the sound waves into kinetic energy.

Nevertheless, the prepared foams are characterized by a relatively high apparent density for sound-absorbing materials, which noticeably affects the portion of pores inside the material, where the sound suppression effect occurs. Therefore, the impact of the structural parameters on the sound absorption performance is not very significant. Generally, the sound absorption performance of the prepared foamed composites does not allow for classifying them as a sound-absorbing material dedicated to the acoustic insulation of buildings [91].

**Figure 7:**
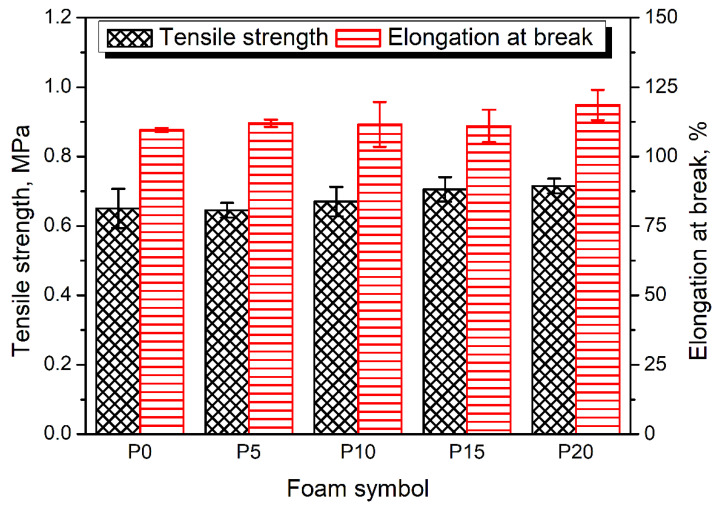
Results of static tensile tests performed for prepared samples.

Figure 7 presents the results of performed static analysis of the prepared PU/GTR composite foams. Incorporating the GTR particles caused a slight enhancement of foams’ tensile performance. It can be associated with the increase in apparent density but also points to the satisfactory interfacial adhesion between PU matrix and GTR particles. Moreover, Table 4 indicates the reduction in the average cell size, which caused stress dispersion and decreased stress concentration, enhancing foams’ strength [92,93]. Such an effect could be one of the components contributing to the slight enhancement of tensile strength. A similar phenomenon was responsible for slightly increasing elongation at break with GTR content. Moreover, the above-mentioned attraction of the isocyanate groups by the hydroxyls present on the surface of the introduced rubber particles yielded a reduced crosslinking of the PU structure, which beneficially impacted its ductility.

**Figure 8:**
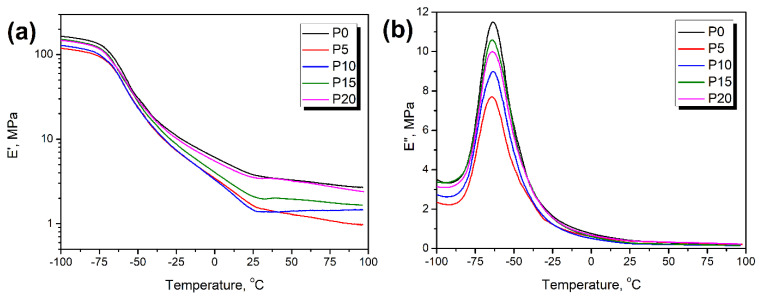
Temperature plots of (**a**) storage modulus and (**b**) loss modulus for prepared foams.

The results of the dynamic mechanical analysis of the prepared PU/GTR composites are presented in Figure 8. It can be seen that the incorporation of the rubber particles reduced the storage modulus of the unfilled foam, especially for the lowest contents of filler. Such an effect could be associated with the disruptions of the PU structures caused by the presence of hydroxyl functionalities on the surface of the GTR particles [59]. However, increasing the filler content led to the enhancement of the foams’ modulus, which can be attributed to the superior performance of the car tires (the primary product from which GTR was produced) compared to the flexible PU foam. For higher GTR loadings, incorporated particles were increasingly more “responsible” for the material’s mechanical performance, so their superior performance overcame the unfavorable effect of the disrupted isocyanate:hydroxyl balance.

Figure 8b, showing the temperature plot of loss modulus, indicated that the damping performance of PU foam was reduced after the introduction of GTR particles. Such an effect indicated that the incorporation of the GTR particles slightly limited the foams’ ability to dissipate mechanical energy, which could be associated with the potential chemical interactions at the interface. A similar effect was noted in previous works dealing with foamed flexible PU/GTR composites [44], as well as in the other works on flexible foamed PU-based composites containing fillers that could chemically interact with isocyanates during foaming [15]. Moreover, the temperature positions of the E” peak can be used to determine the glass transition temperature, which, for all analyzed materials, was in the range from −64.3 °C to −63.7 °C, so the changes were hardly noticeable.

**Figure 9:**
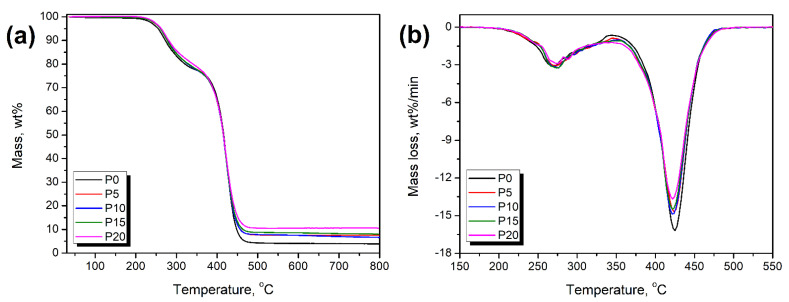
Results of thermogravimetric analysis performed for PU/GTR composite foams and the unfilled PU foam: (**a**) mass loss curves and (**b**) differential thermogravimetric curves.

Figure 9 presents the results of the thermogravimetric analysis of the prepared PU/GTR composites. It can be seen that the incorporation of waste rubber particles did not change the course of the PU thermal decomposition, which was mirrored in the relatively similar appearance of differential thermogravimetric curves. Minor changes in the position of the individual peaks and their magnitude can be attributed to the differences in the chemical composition between the PU and GTR phases. The first peak, around 270 °C, can be attributed to the decomposition of the hard segments, including the urethane bonds. The magnitude of this peak is relatively weak due to the low value of the isocyanate index applied during foams’ preparation. Second, a more potent peak around 422 °C was associated with the decomposition of the soft PU segments, mainly hydrocarbon chains of polyols and styrene–butadiene rubber present in the structure of the GTR [94]. Moreover, the mass loss rate was slightly increased between 340 and 390 °C, which was related to the decomposition of the natural rubber phase of the GTR [46]. Quantitatively, the enhancement of the PU foam thermal stability was noted, which was expressed by the shift of the thermal decomposition onset from 232.5 to 243.6 °C for 20 wt% GTR content. A similar effect was noted in previous work [42] and was attributed to the notably higher stability of the GTR compared to the unfilled foam. Despite the oxidation and partial scission of sulfur bridges during the shredding of the car tires resulting in the generation of GTR, the majority of the crosslinked rubber structure prevailed, maintaining the high thermal stability of the applied particles [95]. The onset of degradation for the GTR applied in the presented work was 256.6 °C, as reported in previous work [42]. Moreover, the char residue after thermal degradation was noticeably higher, mainly due to the high content of carbon black [96].

## 4. Conclusions

The goal of the presented study was the investigation of the impact of applying GTR particles originated from the material recycling of used car tires as a cost-effective waste-based filler for flexible PU foams. Foams were obtained by the two-step prepolymer method instead of the conventional single-step method for better control of the polymerization and volumetric expansion. The introduction of GTR slightly extended the rise time of the foam from the initial 32 s to 33–45 s because of the higher viscosity of the polyol mixture and the attraction of the isocyanate groups by the hydroxyls present on the surface of the rubber particles. The higher viscosity during the volumetric expansion inhibited the dissipation of the heat generated in the exothermic reactions and slightly increased the temperature reached by the foams’ core from 97 °C to 101–104 °C. Nevertheless, for the lower GTR loadings, changes in the foaming kinetics were insignificant due to the contradictory nucleating activity of the solid rubber particles.

Changes in foams’ polymerization induced by GTR were mirrored in their cellular structure. The average cell diameter was reduced from 263.1 µm to 144.8–188.5 µm, which was associated with the above-mentioned nucleating activity of the GTR. The most significant and beneficial reduction was noted for the lowest filler content when the viscosity of the polyol mixture was only slightly elevated. For the higher loadings, the above-mentioned attraction of the isocyanate groups by the hydroxyls present on the surface of rubber particles caused the weakening of the PU matrix, increasing the content of the open cells from 49.0% to 51.9% for 20 pbw of GTR. Cumulatively, the changes in the cellular structure caused a 1.0–4.3% drop in thermal conductivity coefficient, enhancing the thermal insulation performance of the composites compared to the unfilled PU foam. On the other hand, the sound absorption performance of the investigated materials was limited due to the relatively low content of the open cells. It did not allow for classifying them as sound-absorbing materials dedicated to the acoustic insulation of buildings.

Due to the excellent mechanical performance of car tires, the primary application of GTR, the tensile performance of the foams was enhanced despite the disruption of the cellular structure resulting from competitiveness between the hydroxyl groups present in the structure of polyols and on the surface of the GTR particles. Tensile strength and elongation at break were increased by 10 and 8% for 20 parts by weight GTR addition, pointing to the satisfactory interfacial adhesion between the PU matrix and the GTR particles. Moreover, the average cell size reduction caused stress dispersion and decreased stress concentration, enhancing foams’ strength. On the other hand, the dynamic mechanical performance and damping ability of the unfilled foam were reduced after the GTR incorporation due to the weakening of the PU structure.

The application of GTR as filler also enhanced the thermal stability of the foam, shifting the thermal decomposition onset from 232.5 °C to 243.6 °C for 20 pbw GTR content. Such an effect was attributed to the higher stability of the rubber filler compared to the unfilled PU foam, exceeding 256 °C.

Generally, the presented work indicates that GTR can be efficiently applied as a filler for flexible PU foams, which could simultaneously enhance their performance, reduce costs, and limit the environmental impacts due to the application of waste-based material.

## Figures and Tables

**Table 1:** Materials used to prepare flexible PU foams in the presented study.

Material	Producer	Properties/Additional Information
PTMG 2000	DuPont (Wilmington, NC, USA)	Poly(tetramethylene ether)glycol, hydroxyl value—56 mg KOH/g, molar mass—2000 g/mol
Glycerol	Sigma Aldrich (Poznań, Poland)	Hydroxyl value—1800 mg KOH/g
Toluene diisocyanate (TDI)	Sigma Aldrich (Poznań, Poland)	Mixture of 2,4-TDI and 2,6-TDI in the 80/20 ratio
Dabco33LV	Air Products (Allentown, PA, USA)	Catalyst, 33 wt% solution of 1,4-diazabicyclo[2.2.2]octane in dipropylene glycol
Dibutyltin dilaurate (DBTL)	Sigma Aldrich (Poznań, Poland)	Organic tin catalyst
Tegostab B8460	Evonik Industries AG (Essen, Germany)	Foam stabilizer, polyether polydimethylsiloxane copolymer
Distilled water	-	Chemical blowing agent
Ground tire rubber	Grupa Recykl S.A. (Śrem, Poland)	Average particle size—0.6 mm

**Table 2:** Formulations of PU foams analyzed in the presented study.

	Component	Foam Symbol				
		P0	P5	P10	P15	P20
		Content, pbw				
1st step	PTMG	27.03				
	TDI	30.09				
	Total prepolymer	57.12				
2nd step	PTMG	36.80				
	Glycerol	5.11				
	33LV	0.31				
	DBTL	0.27				
	B8460	0.20				
	Water	0.20				
	GTR	0	5	10	15	20

**Table 3:** Composites’ appearance and parameters describing it in quantitative terms.

Sample	Color Parameters						Digital Color Reproduction	Foam Skin Image	Foam Core Image
	L*	a*	b*	ΔE	Chroma	Hue			
P0	86.25	−0.27	3.46	0.00	3.47	−85.54		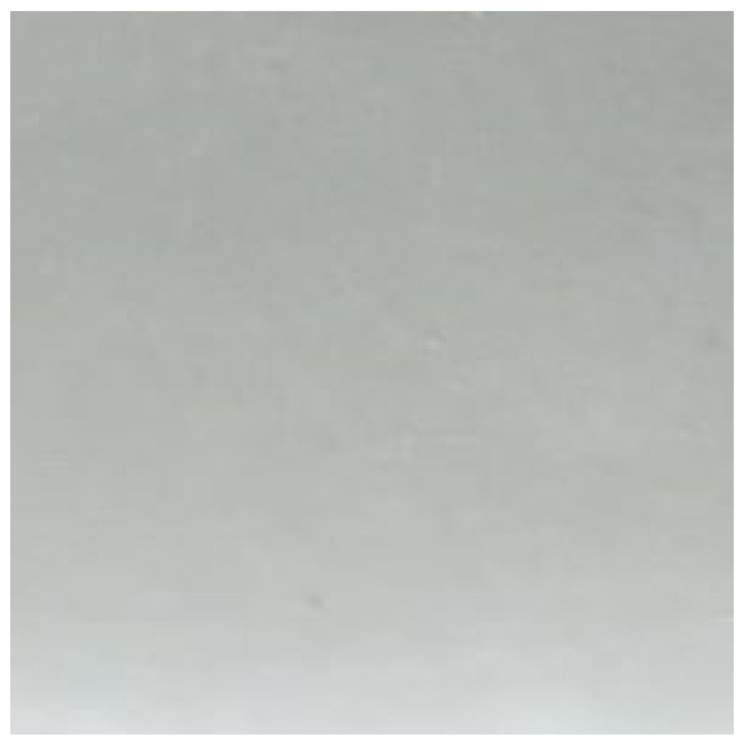	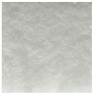
P5	69.80	−0.08	2.71	16.47	2.71	−88.31		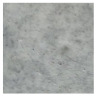	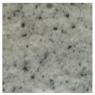
P10	65.40	−0.39	3.36	20.85	3.38	−83.38		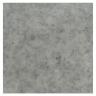	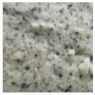
P15	61.97	−0.51	3.33	24.28	3.37	−81.29		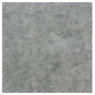	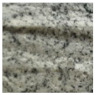
P20	58.47	0.08	5.41	27.85	5.41	89.15		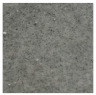	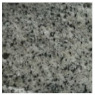

**Table 4:** Parameters describing cellular structure and thermal insulation performance of prepared foamed PU/GTR composites.

Parameter	P0	P5	P10	P15	P20
Average cell diameter, µm	263.1 ± 64.9	188.5 ± 49.7	144.8 ± 87.8	170.2 ± 78.6	178.3 ± 109.0
Circularity	0.48 ± 0.06	0.45 ± 0.17	0.43 ± 0.22	0.39 ± 0.21	0.35 ± 0.20
Aspect ratio	1.32 ± 0.28	1.35 ± 0.30	1.38 ± 0.44	1.42 ± 0.44	1.48 ± 0.41
Roundness	0.79 ± 0.14	0.77 ± 0.14	0.78 ± 0.17	0.75 ± 0.17	0.72 ± 0.16
Open cell content, %	49.00 ± 2.53	53.23 ± 1.97	49.09 ± 2.11	50.14 ± 2.01	51.92 ± 2.66
Apparent density, kg/m^3^	311.8 ± 4.3	297.0 ± 1.4	309.5 ± 1.9	313.0 ± 7.2	322.9 ± 3.4
Sol fraction content, wt%	1.35 ± 0.02	1.91 ± 0.08	2.18 ± 0.32	2.35 ± 0.30	2.74 ± 0.21
Thermal conductivity coefficient, mW/(m·K)	62.90 ± 1.89	61.22 ± 1.84	62.23 ± 1.56	62.27 ± 1.71	60.20 ± 1.51

## Data Availability

The data presented in this study are available in Comprehensive enhancement of prepolymer-based flexible polyurethane foams’ performance by introduction of cost-effective waste-based ground tire rubber particles.
